# Transcriptome analysis reveals potential marker genes for diagnosis of Alzheimer’s disease and vascular dementia

**DOI:** 10.3389/fgene.2022.1038585

**Published:** 2022-11-24

**Authors:** Li Wang, Chunjiang Yu, Ye Tao, Xiumei Yang, Qiao Jiang, Haiyu Yu, Jiejun Zhang

**Affiliations:** ^1^ Department of Geriatrics, The Second Affiliated Hospital of the Harbin Medical University, Harbin, China; ^2^ Department of Neurology, The Second Affiliated Hospital of the Harbin Medical University, Harbin, China; ^3^ Department of Neurology, The First Hospital of SuiHua City, Suihua, China; ^4^ Department of Cardiovascularology, The Fifth Hospital of the Harbin City, Harbin, China; ^5^ Department of Neurology, The Fifth People’s Hospital of the Dalian City, Dalian, China; ^6^ Rehabilitation Department of Jiamusi Center Hospital, Jiamusi, China; ^7^ Department of Neurology, Hebei Yanda Hospital, Hebei, China

**Keywords:** Alzheimer’s disease, vascular dementia, potential mechanism, WGCNA, immune cell infiltration

## Abstract

Alzheimer’s disease (AD) and vascular dementia (VD) are the two most common forms of dementia, share similar symptoms, and are sometimes difficult to distinguish. To investigate the potential mechanisms by which they differ, we identified differentially expressed genes in blood and brain samples from patients with these diseases, and performed weighted gene co-expression network analysis and other bioinformatics analyses. Weighted gene co-expression network analysis resulted in mining of different modules based on differences in gene expression between these two diseases. Enrichment analysis and generation of a protein-protein interaction network were used to identify core pathways for each disease. Modules were significantly involved in cAMP and AMPK signaling pathway, which may be regulated cell death in AD and VD. Genes of cAMP and neurotrophin signaling pathways, including *ATP1A3*, *PP2A*, *NCEH1*, *ITPR1*, *CAMKK2*, and *HDAC1*, were identified as key markers. Using the least absolute shrinkage and selection operator method, a diagnostic model for AD and VD was generated and verified through analysis of gene expression in blood of patients. Furthermore, single sample gene set enrichment analysis was used to characterize immune cell infiltration into brain tissue. That results showed that infiltration of DCs and pDCs cells was increased, and infiltration of B cells and TFH cells was decreased in the brain tissues of patients with AD and VD. In summary, classification based on target genes showed good diagnostic efficiency, and filled the gap in the diagnostic field or optimizes the existing diagnostic model, which could be used to distinguish between AD and VD.

## Introduction

Damage to neuronal structure may cause loss of nervous system function, which can lead to neurodegenerative disease. Alzheimer’s disease (AD) is one of the most common neurodegenerative diseases worldwide (Lane et al., 2018). Alzheimer’s disease is characterized by the presence of extracellular amyloid plaques caused by abnormal APP processing, resulting in β-amyloid peptide aggregation (Calsolaro and Edison, 2016). There is no cure for AD, disease progression cannot be reversed, and symptoms gradually worsen until patients lose their ability to care for themselves. Given the prolonged course of disease progression, AD results in incredible suffering for patients and their families, and places an enormous burden on healthcare systems. Previous studies have confirmed that the pathogenesis of AD can include genetic factors. Mutations in *APP*, *PSEN1*, and *PSEN2* have been shown to play key roles in familial AD (Lanoiselee et al., 2017). Advances in human disease research have shown that many complex diseases are caused by multiple genes. These genes interact to form a network that collectively influences the pathogenesis of diseases (Ding et al., 2019). Therefore, gene set risk assessment is viewed as a more accurate and effective method to study the genetic basis and mechanisms of complex diseases.

Vascular dementia (VD) is also a common form of dementia. The symptoms of VD are similar to those of AD, which can often complicate differential diagnosis (Uwagbai and Kalish, 2022). In some patients, VD and AD may coexist, resulting in a pathological condition known as mixed dementia. The etiology of dementia is complex, and treatment is difficult. To data, these biomarkers play a vital role for diagnosis and prognosis of AD or VD. Studies have demonstrated that identifying REPS1 as a candidate therapeutic biomarker in AD and VD (Luo et al., 2022). RBM8A (Zou et al., 2019) and YKL-40 (Mavroudis et al., 2021) were significantly associated with AD pathophysiology. Furthermore, toll-like receptor 2 (TLR2) is the hub gene that may participate in the course of VD (Wang et al., 2022). Therefore, exploration of the pathogenesis and biomarkers of VD/AD-induced dementia could deepen understanding of dementia, which may aid in diagnosis and improve choice of treatment strategies.

Studies have indicated that the multifactorial pathophysiology of dementia is not restricted to neuronal cells, and the immune system may play a key role (Heneka et al., 2015). For example, during the AD onset, T lymphocytes may infiltrate into brain tissue *via* the choroid plexus and participate in adaptive immune response. CD8 T lymphocytes were detected in the cerebrospinal fluid (CSF) of 11 patients with AD (Lueg et al., 2015). Other types of immune cells, including monocytes, macrophages, neutrophils, and T cells from the peripheral blood, were found to be broadly involved in the pathogenesis of AD (Polfliet et al., 2001; Ziegler-Heitbrock, 2007; Baik et al., 2014; Gate et al., 2020). Moreover, differences in levels of lymphocyte subsets were found in the brains of patients with different types of dementia, and a significant increase in classical natural killer (NK) cells was observed in VD (D'Angelo et al., 2020).

In this study, the expression profile of brain tissue samples from the Gene Expression Omnibus (GEO) data set and the blood expression profile of 3 patients with AD, 6 patients with VD, and 3 healthy donors were analyzed. The Weighted Gene Co-Expression Network Analysis (WGCNA) method and least absolute shrinkage and selection operator (LASSO) model were used to establish diagnostic gene signatures for AD and VD, and to identify potential therapeutic targets. Furthermore, we performed single-sample gene set enrichment analysis (ssGSEA) to quantify immune cell infiltration to provide a theoretical foundation for further research.

## Methods and materials

### Data collection and processing

**FIGURE 1:**
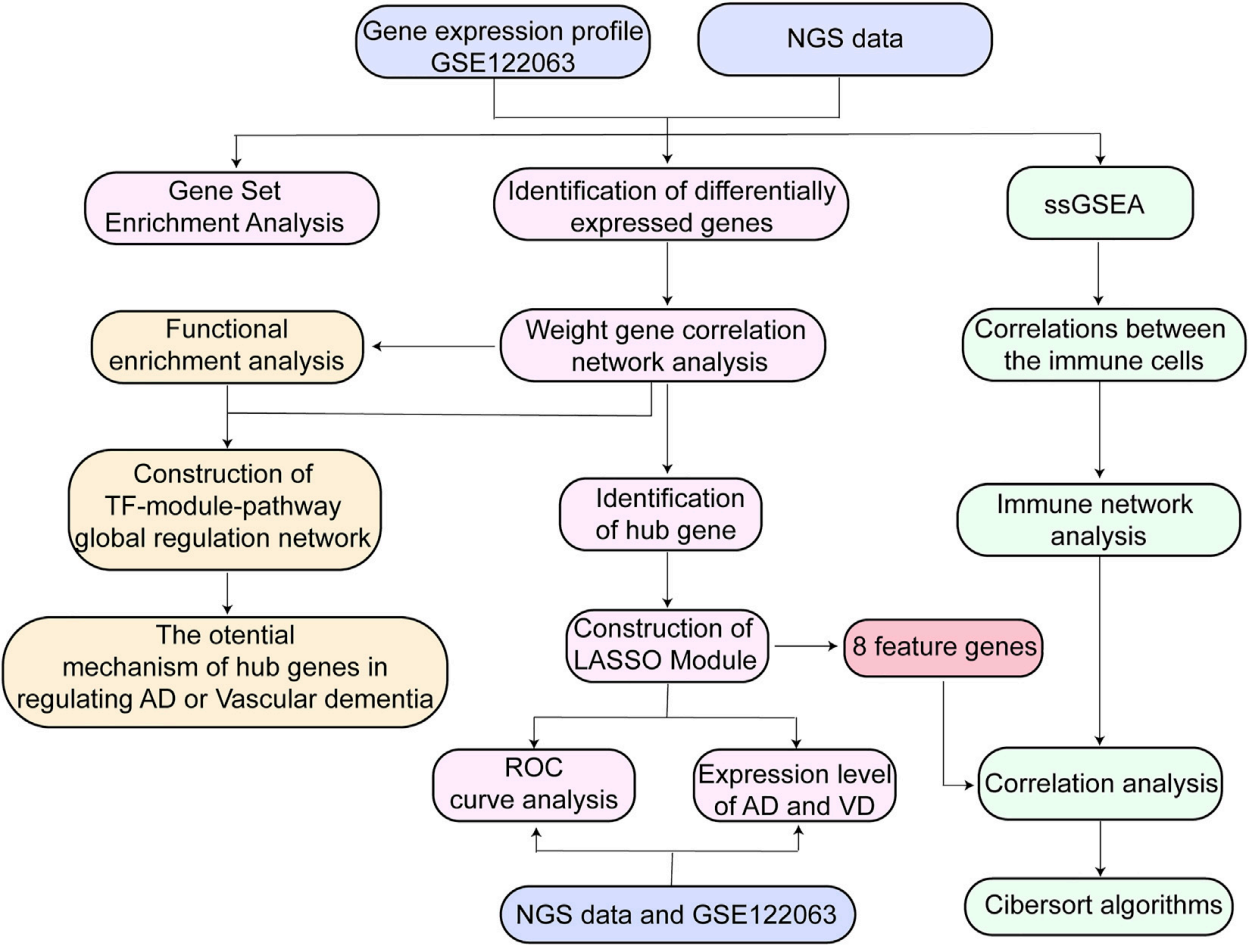
Flow chart of study design. AD, Alzheimer’s disease; LASSO, least absolute shrinkage and selection operator; NGS data, Next Generation Sequencing data; ROC, receiver operating characteristic curve; ssGSEA, Single Sample Gene Set Enrichment Analysis; TF, transcription factor; VD, vascular dementia.

High-throughput RNA Sequencing data were used to construct the blood RNA expression profiles of 3 patients with AD, 6 patients with VD, and 3 healthy donors. The AD and VD samples related clinical information were shown in Table 1. Public dataset GSE122063 was obtained from the GEO database (https://www.ncbi.nlm.nih.gov/geo/query/acc.cgi?acc=GSE122063), which includes brain samples from 56 individuals with AD (44 female and 12 male), 36 individuals with VD (16 female and 20 male), and 44 healthy individuals (24 female and 20 male). The range of age was 60–91 years for healthy controls, 62–96 years for VD patients and 63–91 years for AD patients. Gene expression profiling was performed on frontal and temporal cortex tissue from patients with VD and AD, and healthy controls obtained from the University of Michigan Brain Bank. Controls and patients with AD had no infarcts in the autopsied hemisphere. In order to unify the different data, the normalizeBetweenArrays function in the limma package (Ritchie et al., 2015) was used to normalize the gene expression profiles. If a gene corresponds to multiple probes, the average expression value of these probes was chosen as the expression value of the gene. The workflow of the present study was shown in Figure 1.

**TABLE 1:** Clinical information of the sequencing data.

Library name	Sex	Age	Disease type	Blood pressure	Blood homocysteine	C Reactive protein	Fasting blood-glucose	Cholesterol total	Glycerin trilaurate	High density lipoprotein cholesterol	Low density lipoprotein cholesterol
A-D1	Woman	86	AD + DM	120/70	10.29	14.17	6.73	5.2	1.5	1.52	2.9
V1	Woman	83	VD	130/80	10.46	1.47	6	4.61	1.38	1.21	2.76
V2	Woman	83	VD + DM	165/89	11.16	1.15	5.51	2.52	0.71	1.29	0.83
V-D1	Woman	84	AD	152/88	9.01	10.89	7.5	4.88	2.22	1.14	2.93
A1	man	87	AD + DM	94/57	14.03	7.44	5.9	5.68	4.3	0.89	2
A-D2	man	76	VD + DM	160/100	13.83	9.93	7.48	5.26	2.16	1.26	3.86
V3	man	50	VD + DM	150/90	10.09	1.63	4.19	3.38	1.44	1.01	1.6
V-D2	man	73	VD	146/89	19.92	5.43	7.74	4.54	8.73	0.76	1.43
V7	Woman	61	VD	146/78	9.75	1.98	6.79	4.46	1.33	0.99	2.75

All analyses in this study were based on the Bioinforcloud platform (http://www.bioinforcloud.org.cn), including expression profiles of GSE122063 and NGS data analysis. Bioinforcloud platform is a self-developed bioinformation analysis platform, which is a collection of data download, analysis and visualization of results, brought together various data processing and bioinformatics analysis methods. Furthermore, the DEGs were screened in two comparion-pairs (AD or VD compared to normal tissues in GSE122063 dataset and NGS data), which adjusted P values >0.05 were significant.

### RNA purification and library preparation

Total RNA was extracted and a library was prepared according to the reagent manufacturer’s instructions. RNA purity was verified using a kaiaoK5500®Spectrophotometer (Kaiao, Beijing, China), and the integrity of the RNA was evaluated using an RNA Nano 6000 Assay Kit on an Agilent 2,100 Bioanalyzer (Agilent Technologies, Palo Alto, CA, USA). Two micrograms of total RNA from each sample was used as the input for library construction using a NEBNext^®^ Ultra™ RNA Library Prep Kit for Illumina^®^ (#E7530L, NEB, USA).

### Library clustering and sequencing

Clustering was performed on the HiSeqPE Cluster reagent kit v4-cBot-HS (Illumina) on the HiSeqPE cluster generation system according to the manufacturer’s instructions. After clustering generation, they were sequenced on the Illumina platform of the library, and 150bp paired-end reads were generated.

### Differential expression analysis

The expression profiles of 12 cases from the GSE122063 were selected using the Intersect function (Chen and Boutros, 2011) for analysis of co-expressed genes. Differentially expressed genes (DEGs) between AD and VD were screened using the limma package in R (Ritchie et al., 2015). Genes with adjusted *p* < 0.05 were considered to be significantly differentially expressed.

### Identification of hub genes using WGCNA

To find co-expressed gene modules, we extracted DEGs from GSE122063 to perform co-expression network using WGCNA package in R (Langfelder and Horvath, 2008). First, the “dist” function was used to calculate the distance between the variables, and a hierarchical clustering analysis was performed using the “hclust” function. We calculated the power parameters using the “pickSoftThreshold” function, which in turn assessed the average connectivity and independence between the modules. The power is deemed proper when the independence exceeds 0.9. Co-expressed gene modules were identified by dynamic tree cutting methods, and hierarchical clustering was established. Subsequently, we calculated module-disease correlation using Pearson correlation analysis to obtain relevant modules with disease status (AD; VD). Furthermore, associations between genes and modules were defined as module memberships (MM), and gene significance (GS) was determined by a combination of phenotypic feature information and the co-expression. A gene was defined as a hub gene in the module if it had GS > 0.2 and MM > 0.9.

### Functional enrichment analysis

The module eigengene were analyzed using Gene Ontology (GO) function and Kyoto Encyclopedia of Genes and Genomes (KEGG) pathway enrichment analysis by the clusterProfiler package (Yu et al., 2012) in R. Results with *p* < 0.05 were considered significant. We screened biological processes (BP) and KEGG pathways related to AD or VD using Gene Set Enrichment Analysis (GSEA) (Subramanian et al., 2005) by the MSigDB c2.cp.kegg.v7.2.symbols.gmt gene set collection (Liberzon et al., 2015), P value <0.05 with the pathways were considered statistically significant.

### Gene set variation analysis

We performed gene set variation analysis (GSVA) of the expression profile data sets GSE122063 and NGS data using the GSVA package in R (Hanzelmann et al., 2013). Individual samples were scored with the gene set using GSVA, and GSVA scores were obtained for each sample. The GSVA scores for gene sets were calculated for the GSE122063 and NGS data.

### Construction of the protein–protein interaction network

Based on the interactions of human transcription factor (TFs) with their target genes in the TRRUST v2 database (Li et al., 2018), the hypergeometric test was used to predict potential TFs regulating functional modules. In addition, complex cellular functions were performed through the interactions between proteins. The PPI network was constructed using Cytoscape software (http://cytoscape.org/) (Shannon et al., 2003) according to STRING database (Szklarczyk et al., 2017).

### Construction of LASSO model and receiver operating characteristic curve analysis

We used LASSO as predictive tool to select the best features of high-dimensional data (Ding et al., 2019). We extracted key genes to construct LASSO models to distinguish between AD and VD. Then, we calculated gene expression values for the regression coefficient, in which the formula weighted the expression of gene expression: signature index = ExpGene1*Coef1 + ExpGene2*Coef2 + ExpGene3*Coef3+. …"Coef” is the regression coefficient of the gene, “Exp” represents the expression value of the gene. In addition, we randomly assigned samples in the GSE122063 dataset to the training set (75%) and to the test set (25%). To verify that the LASSO model could discriminate between AD and VD, ROC curve analysis was performed on the training and test sets using pROC package (Robin et al., 2011). To further validate the diagnostic efficiency of LASSO model, we validated the results in sequencing data.

### Prediction of miRNA-target gene interactions

Interactions between the top 3 miRNAs with the largest |log FC| values and target genes were predicted using the TargetScan (Http://www.targetscan.org/vert_72) database (Lewis et al., 2005). Cytoscape software (Shannon et al., 2003) was used to visualize the network.

### Single-sample gene set enrichment analysis

Relative immune cell infiltration levels in single sample were quantified using ssGSEA in R package GSVA (Hanzelmann et al., 2013). The degree of infiltration of the immune cells in the AD and VD samples were determined. Immunity network analysis was used to explore the correlation between immune cells. We also determined the correlation between feature genes and immune infiltration. The CIBERSORT algorithm (https://cibersort.stanford.edu/) was used to infer cell type proportions in the data from AD samples.

## Results

### Identification of differentially expressed gene in AD and VD

**FIGURE 2:**
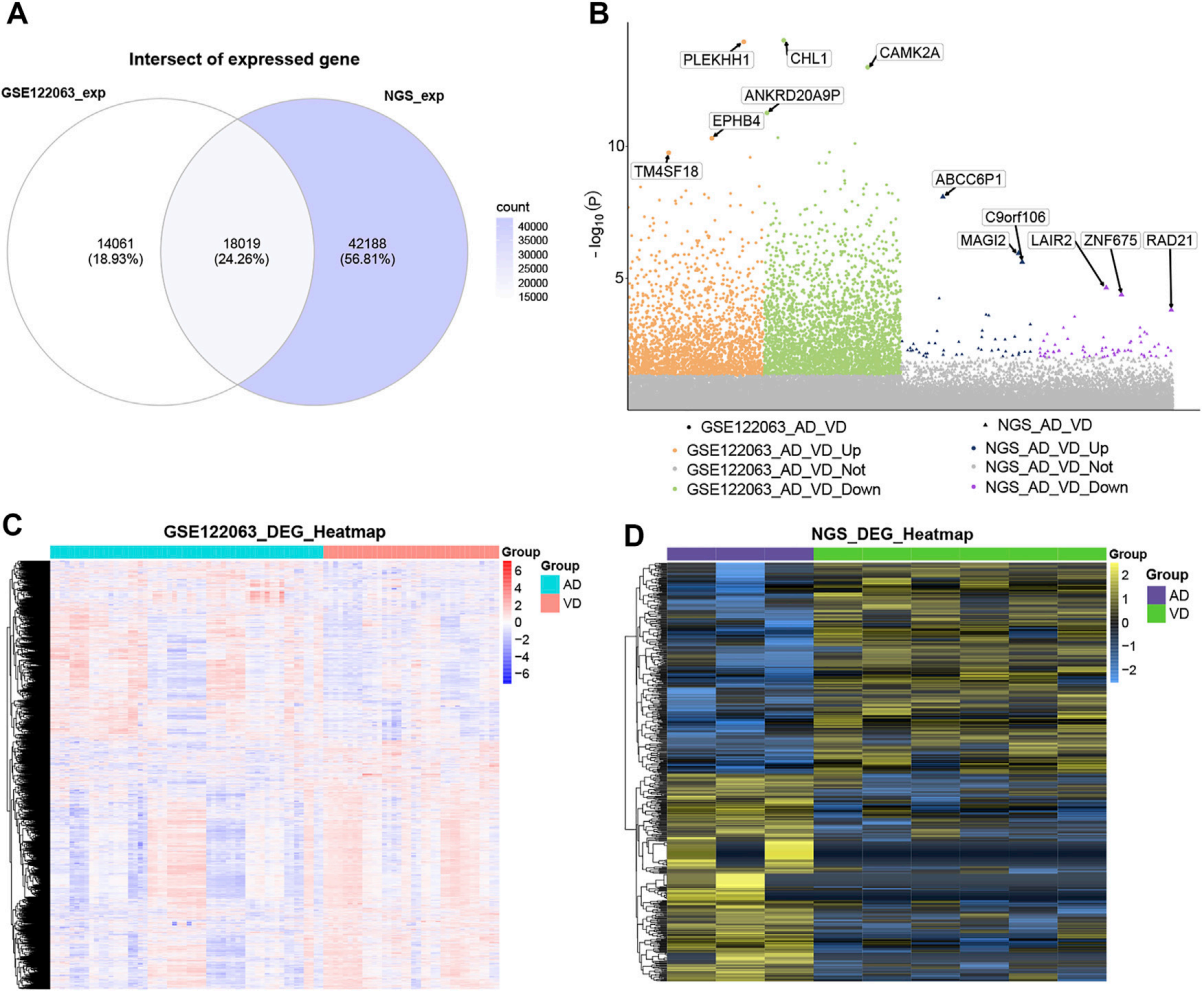
Differential expression analysis. **(A)** The venn diagram showed the genes overlaped in the blood expression profile of GSE122063 data set and sequencing data. **(B)** Manhattan map of differential gene expression. **(C)** The heat map of DEGs in GSE122063 data set. **(D)** The heat map of DEGs in Next Generation Sequencing data (NGS). AD, Alzheimer’s disease; VD, vascular dementia.

A total of 18,019 overlapping genes were detected between the profiles in the GSE122063 data set and our sequencing data (Figure 2A). These overlapping genes were further used for differentially expressed gene (DEG) analysis (Figure 2B). In the GSE122063 data set, there were 5,340 DEGs observed between the AD and VD groups, including 2,234 up-regulated genes and 3,106 down-regulated genes. In our NGS sequencing data set, there were 587 DEGs between the AD and VD groups, including 291 up-regulated genes and 296 down-regulated genes. These DEGs were able to discriminate between AD and VD (Figures 2C,D).

### Gene modules associated with AD or VD

**FIGURE 3:**
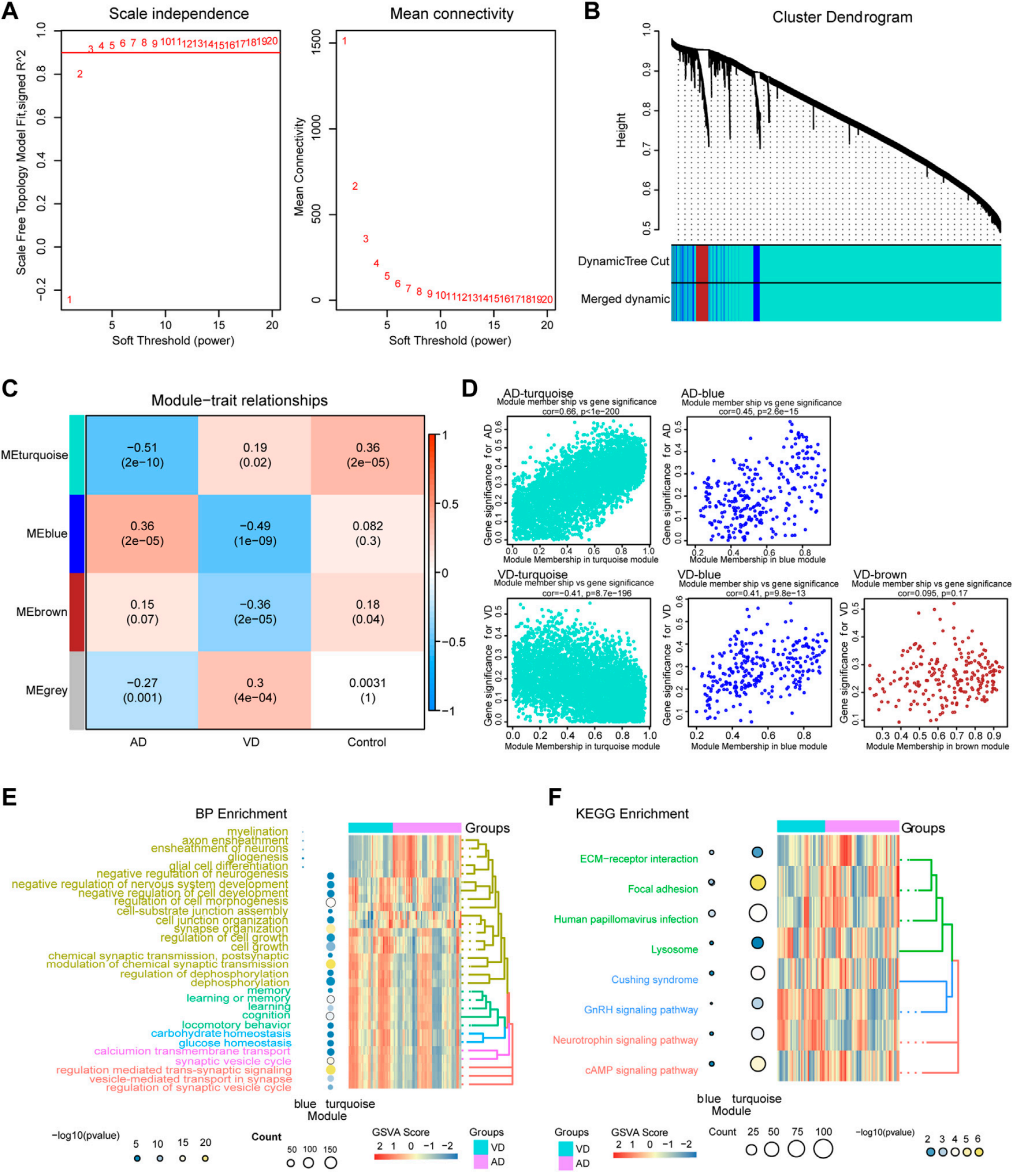
Weighted Gene Co-Expression Network Analysis. **(A)** Definition of power related to modules. **(B)** Recognition module. **(C)** The turquoise module negatively correlated with AD and positively correlated with VD, blue module was positively correlated with AD, but the opposite of VD, brown module was negatively correlated with VD. Red, positive correlstion; Blue, negative correlation. **(D)** Module membership and gene significance strongly correlated with each other within each module. **(E)** Biological processes involving genes of the different modules. **(F)** KEGG pathways involving genes of the different modules. AD, Alzheimer’s disease; VD, vascular dementia; KEGG, Kyoto Encyclopedia of Genes and Genomes.

The WGCNA method was used to identify the core gene modules that differentiated between AD and VD (Figure 3). The results showed that when the minimum power was 3, the independence was greater than 0.90 (Figure 3A). As shown in Figure 3B, we identified four key gene modules that discriminated between AD and VD. The turquoise module was negatively correlated with AD and positively correlated with VD (r = -0.51, P = 2e-10 for AD and r = -0.19, *p* = 0.02 for VD). The blue module positively correlated with AD, but negatively correlated with VD (r = 0.36, P = 2e-05 for AD and r = -0.49, P = 1e-09 for VD). The brown module negatively correlated with VD (coefficient = -0.36, P = 2e-05) (Figure 3C). In the turquoise module, using GS AD > 0.2 and MM > 0.9 as thresholds, 292 genes were identified as up-regulated hub genes in AD and 24 genes were identified as down-regulated hub genes in VD. In the blue module, using GS AD > 0.2 and MM > 0.9 as thresholds, 4 genes were identified as up-regulated hub genes in AD and 5 genes were identified as down-regulated hub genes in VD. In the brown module, using GS > 0.2 and MM > 0.9 as thresholds, 8 hub genes were identified as poorly expressed in VD (Supplementary Table S1). The hub genes in the turquoise and blue modules were associated with both AD and VD, and hub genes in the brown module was associated with VD (Figure 3D).

Module genes functional enrichment analysis showed that turquoise module genes were significantly involved in biological processes related to neurotransmitters and synaptic regulation such as modulation of chemical synaptic transmission, regulation of trans-synaptic signaling, synapse organization, and vesicle-mediated transport in synapse. The blue module genes were significantly involved in biological processes related to glial cells and nerve sheath cells such as myelination, glial cell differentiation, ensheathment of neurons, and glial cell differentiation. The two modules were associated with KEGG pathways related to cAMP signaling pathway, neurotrophin signaling pathway, GnRH signaling pathway, and ECM- receptor interaction. (Figures 3E,F). Above all, the pathways of module genes may be play a vital role and that promote the development and progress in AD or VD.

### Validation of critical pathways in AD and VD

**FIGURE 4:**
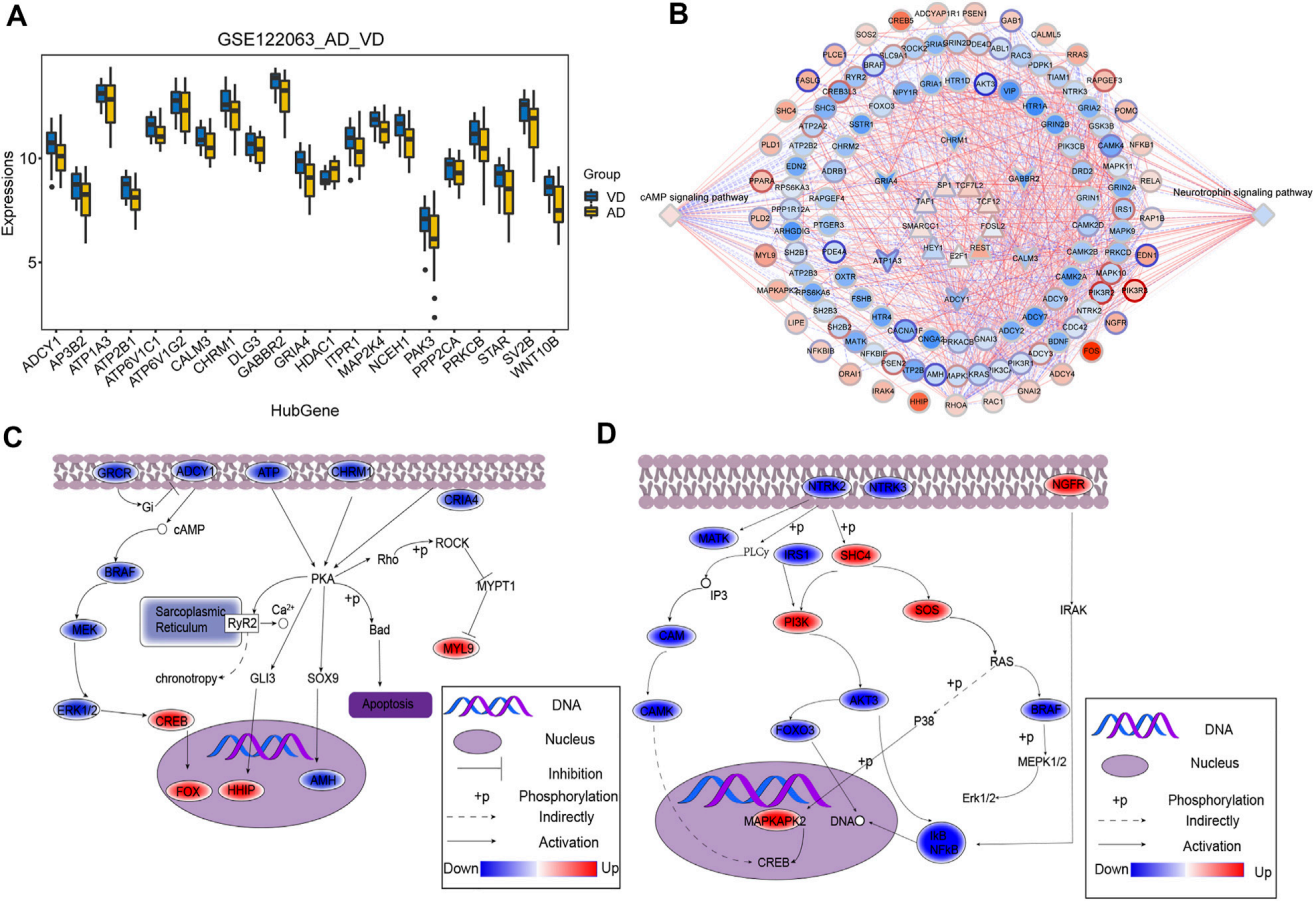
Integrated regulation of AD or VD by change in hub gene expression of modules. **(A)** The expression of target genes in the GSE122063 data set. The thick black bar in the middle indicates the interquartile range, and the black line extending from it represents the 95% confidence interval. **(B)** Integrated regulatory network of cAMP signaling pathways and Neurotrophin signaling pathways. **(C,D)** Maps of gene-pathway correlations in both blue and turquoise module. AD, Alzheimer’s disease; VD, vascular dementia.

The overlapping genes among the hub genes and the genes identified in KEGG pathway analysis were evaluated further (Supplementary Table S2). A total of 21 hub genes were selected as target genes in the data set (Figure 4A). Then, we constructed a TF-module genes-pathway global regulation network containing 9 TFs and 6 hub genes (Figure 4B). Finally, the mechanisms of different modules in progression of AD or VD were explored (Figures 4C,D).

### LASSO model can predict AD and VD

**FIGURE 5:**
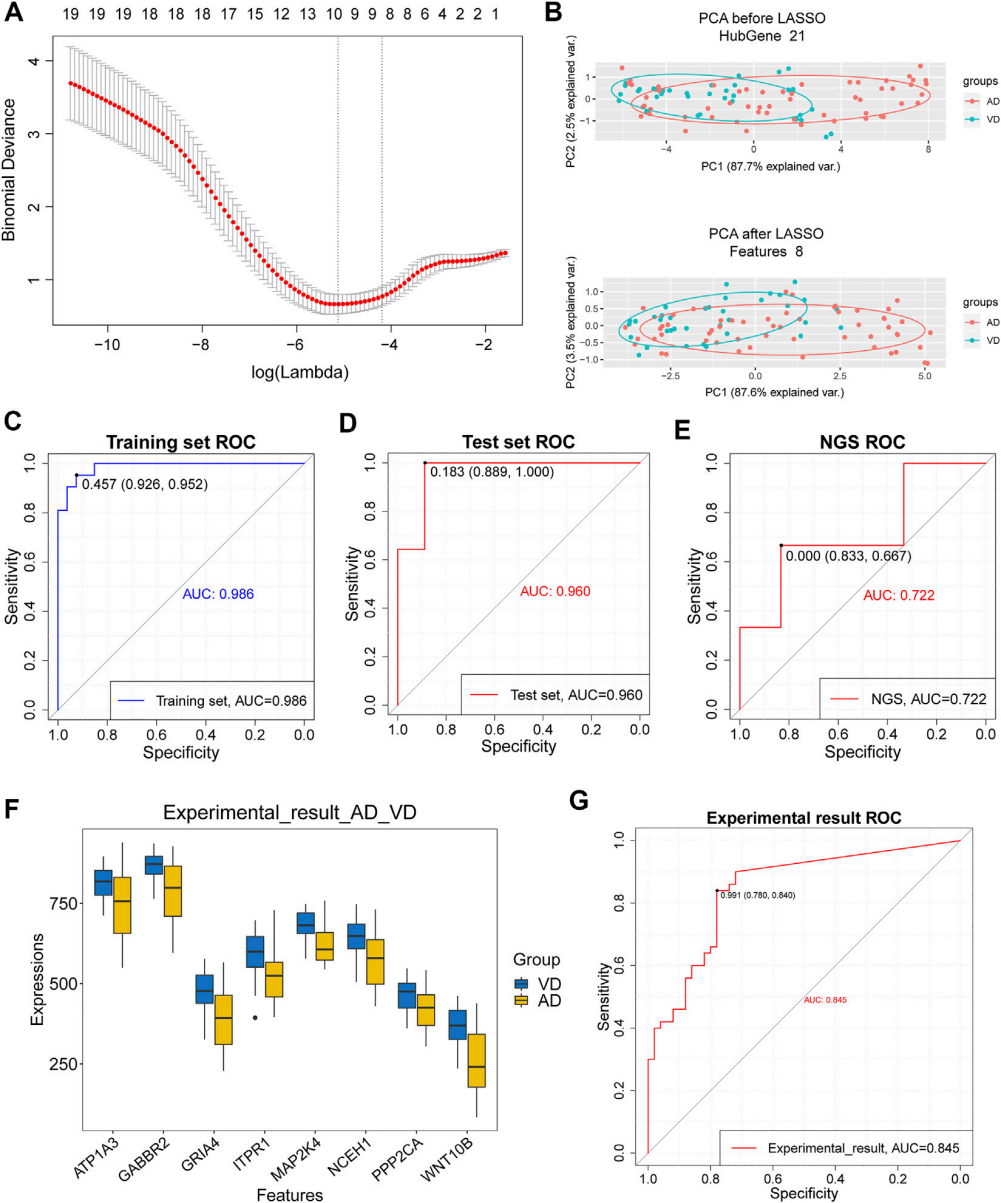
Assessment of models for identification of Alzheimer’s disease (AD) and vascular dementia (VD). **(A)** 10-fold cross-validation for tuning parameter selection in the LASSO model. **(B)** PCA prior to and after LASSO variable reduction. LASSO, least absolute shrinkage and selection operator; PCA, principal component analysis. **(C,D)** ROC curve for patients with AD and patients with VD in the training and test sets. **(E)** ROC curve for patients with AD and patients with VD in the NGS dataset. **(F)** Gene expression levels in patients with AD and patients with VD in the NGS and GSE122063 data sets. The thick black bar in the middle indicates the interquartile range, and the black line extending from it represents the 95% confidence interval. **(G)** ROC curves for patients with AD and patients with VD in the NGS and GSE122063 data sets.

Eight target genes were identified with non-zero regression coefficients as optimal features from 21 target genes in the training set using the LASSO method and 10-fold cross-validation (Figure 5A). Principal component analysis (PCA) showed that the target genes could distinguish AD from VD (Figure 5B). The accuracy of the 8 feature genes based on LASSO model was 0.986 in the training set and 0.960 in the test set. This demonstrated that the model was robust (Figures 5C,D). The results using our sequencing data agreed with the results from the GSEA dataset (Figure 5E). Moreover, the expression of the 8 feature genes was significantly higher in patients with VD than in patients with AD (Figure 5F). The accuracy of the 8 feature genes for discrimination between AD and VD was 0.845 (Figure 5G). In conclusion, we screened 8 feature genes for discrimination of AD and VD by LASSO model, including WNT10B, PPP2CA, NCEH1, MAP2K4, ITPR1, GRIA4, GABBR2 and ATP1A3.

### Immune cells infiltration in AD and VD

**FIGURE 6:**
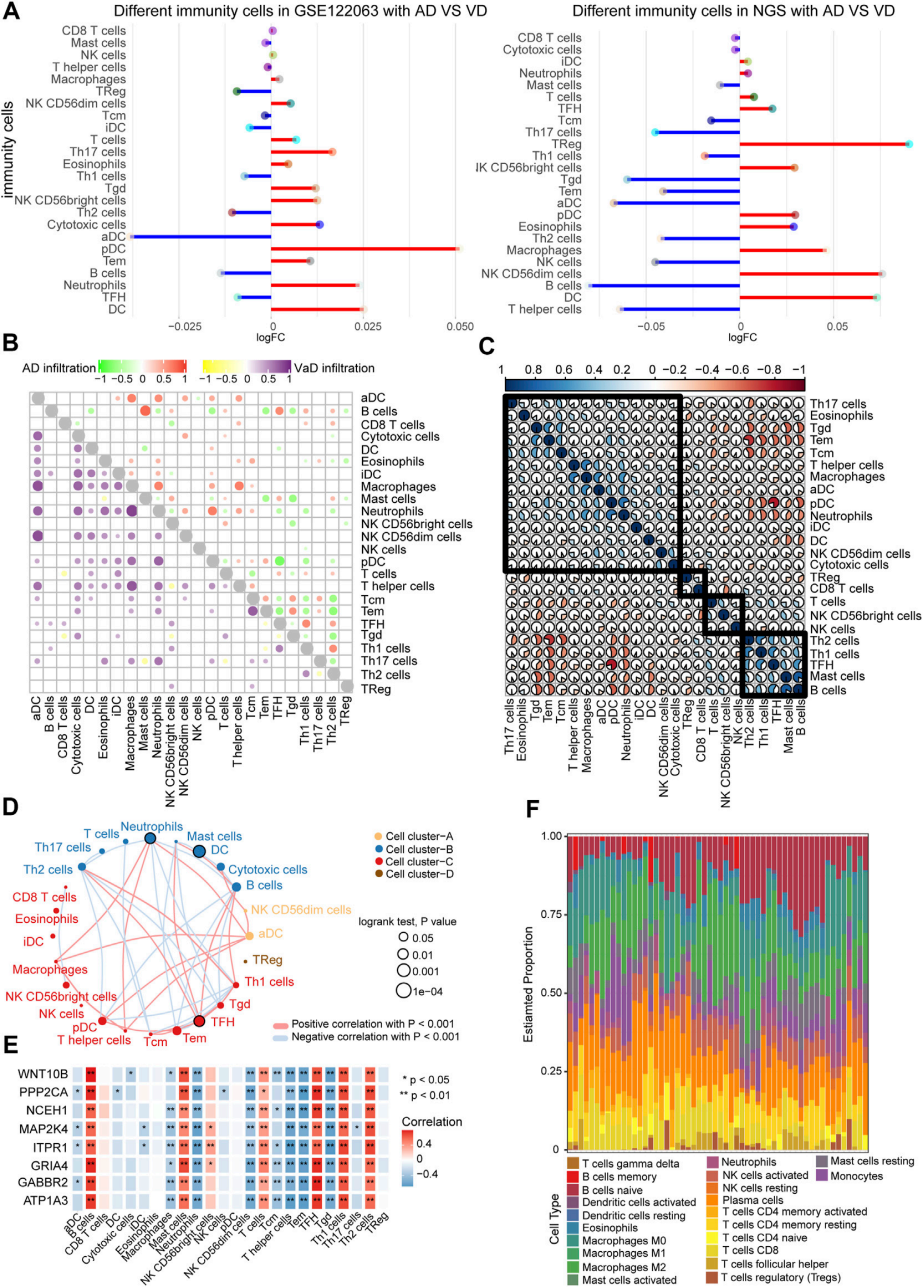
Correlation between immune cells in AD and VD. **(A)** Expression of differentially abundant immune cells in the GSE122063 and NGS datasets. **(B)** Correlation between immune cell types in AD and VD. Red and purple represent positive correlations, and green and yellow indicate negative correlations. **(C)** Correlation between immune cells. The blue section indicates activation, and the orange section indicates inhibition. **(D)** Network of immune cell types (abundance). Circles represents the prognostic effect of the cell type, and the thickness of the line indicates the strength of correlations between the cell types. **(E)** Correlation between immune cell types and the eight featured genes. **(F)** Estimated proportions of immune cell types in AD. AD, Alzheimer’s disease; VD, vascular dementia.

The GSE122063 data and our NGS data were used to investigate the immune cell types in the AD and VD samples. Dendritic cells (DCs) and plasmacytoid dendritic cells (pDC) were present at significantly greater levels in the AD and VD samples (Figure 6A). Correlation analysis between the 24 immune cell types showed that increased infiltration of B cells was significantly correlated with AD, and infiltration of aDCs was significantly correlated with VD (Figure 6B). We performed correlation analysis on immune cells using CIBERSORT (proportion). The results showed that pDCs were positively associated with neutrophils (Figure 6C). In addition, we also clustered immune cells based on abundance, resulting in four clusters (Figure 6D). As shown in Figures 6B,E cells and T follicular helper (TFH) cells were correlated with seven featured genes. We found that plasma cells represented the highest proportion of infiltrated immune cells (Figure 6F).

## Discussion

Progress in modern biotechnology and big data analysis has resulted in expansion of biomedical research of diseases beyond clinical symptoms and manifestations. Research has increasingly targeted the regulatory mechanisms of diseases at the molecular level. Previous studies have shown that onset and progression of AD were not caused by a single gene or a few mutations, but by disruption of a comprehensive gene regulation network (Raikwar et al., 2018; Ding et al., 2019).

In this study, using module mining analysis of data sets, we built a module-related biological network. Module mining using WGCNA resulted in identification of three modules associated with AD and VD. Among these, turquoise module genes were up-regulated in AD and down-regulated in VD. Blue module genes were down-regulated in AD and up-regulated in VD. Brown module genes were down-regulated in VD. The results showed that the blue module was enriched in biological processes associated with glial cells and nerve sheath cells, and the turquoise module was associated with cell cycle, synapse, and neurotransmission. Abnormal glial cell function has been shown to play an important role in the pathophysiology of AD (Herculano-Houzel, 2014). Under certain conditions, microglia express proinflammatory factors that may accelerate development of AD (Heppner et al., 2015). In AD, oxidative damage results in changes in cell cycle regulation. Cell cycle dysfunction may play an important role in neuronal dysfunction in AD, and may represent a potential therapeutic target (Bonda et al., 2010). These findings have been shown to be associated with development of AD and VD (Ding et al., 2019). In general, the genes of modules were involved in pathways play a vital role and which may promote the occurrence of disease course in AD and VD.

We identified six hub genes that regulate key cellular signaling pathways. According to previous studies, ATP1A3 (Shrivastava et al., 2020), PP2A (Wang et al., 2019), NCEH1 (Ding et al., 2019), ITPR1 (Uddin et al., 2018), and CAMKK2 (Sabbir, 2018) have been shown to be associated with onset of AD. The target genes identified in GSE122063 data set were highly expressed in VD, except for HDAC1. In addition, reduced cAMP signaling through PKA has been shown to be a key feature of AD pathology, and local increases in cAMP signaling may contribute to AD pathology (Kelly, 2018). Neurotrophin plays an important role in central and peripheral neuron survival and differentiation. Inhibition of axonal neurotrophin transport may also contribute to development of AD (Wu et al., 2009). Our results showed that 9 TFs regulated these pathways through six interacting hub genes. A comprehensive regulatory landscape network map was constructed. ATP1A3, PP2A, NCEH1, ITPR1, CAMKK2, and

Eight feature genes were identified using LASSO regression that may be involved in development of AD. Studies have shown that the AMPA receptor (GRIA4) was significantly up-regulated in the hippocampus of patients with AD (Jacob et al., 2007). MAP2K4 was exhibited brain-specific gene and to play essential roles in the regulation of cell proliferation in AD (Wu et al., 2021), while MAP2K4 was related with the condition and prognosis of endometrial carcinoma (Zhang et al., 2022). ITPR1 (Seo et al., 2020) and GABBR2 (Yin et al., 2021) may be associated with AD, and prostate cancer (Choi et al., 2022). Furthermore, PPP2CA as a candidate gene that it may affect the risk of AD (Vazquez-Higuera et al., 2011). NCEH1 and WNT10B, and ATP1A3 have been rarely reported to be associated with AD, but WNT10B has an important role in progression of colorectal cancer (Shi et al., 2019) and hepatocellular carcinoma (Zhou et al., 2020). Therefore, suggesting that WNT10B, ITPR1, GABBR2, ATP1A3, NCEH1, MAP2K4, PPP2CA, and GRIA4 may play a vital role in AD and VD, while also need more studies to further validate the expression of hub genes. Furthermore, the LASSO model based on target genes showed good diagnostic value, which was validated using our sequencing data.

Studies have reported that age-related immunoadaptive recombination causes lymphocyte immunity as a whole to begin having a role in an intermediate metastable state, and the dominant role of immune factors in the pathogenesis of VD and AD (Nuvakhova and Rachin, 2020). In the present, to quantify the extent to which the immune cells infiltrated into brain tissue, we used ssGSEA. The results showed that infiltration of B cells and TFH cells was significantly higher in AD and VD. Nuclear factor of activated B cells has been shown to be involved in physiological inflammatory processes, and was a promising target for treatment of AD (Seo et al., 2018). We also detected decreased levels of B and T lymphocytes in AD and VD, though the decreases were not statistically significant (Busse et al., 2017). Follicular helper CD4 T cells are specialized helpers of B cells (Crotty, 2011). Regulatory T cells were significantly reduced in VD patients, and the T cells were significantly increased in AD patients, possibly due to the inflammation triggered by Aβ (Ziegler-Heitbrock, 2007). Recently, neuroinflammation and tissue-resident immune cells are increasingly recognized as key factors in the pathogenesis of AD (Guzman-Martinez et al., 2019; Lutshumba et al., 2021). Therefore, we speculated that immune cell interactions may promote development of AD and VD.

In conclusion, we used WGCNA analysis to mine modules related to AD and VD, and identified target genes that may regulate AD and VD. Using LASSO modeling, we showed that these target genes could distinguish between AD and VD. Furthermore, modules of WGCNA were significantly involved in cAMP signaling pathway, suggesting genes of pathways may be promote the cell death in AD and VD. However, this study had some limitations. First, the study was based primarily on bioinformatics analysis, while the experiments were not validated, so we only offer theoretical conclusions. Second, our sequencing data can discriminate was validated between AD and VD, while the samples size was relatively small, so studies with large sample sizes are warranted to affirm our findings. Therefore, this study provided a theoretical basis for discrimination between AD and VD, and provided new insight for future studies.

## Data Availability

The datasets presented in this study can be found in online repositories. The names of the repository/repositories and accession number(s) can be found in the article/Supplementary Material.

## Supplementary material

Supplementary Table S1 Hub genes.

Supplementary Table S2 21 hub genes regulating KEGG pathway.
